# Impact of maternal age and body mass index on the structure and function of the heart in newborns: a Copenhagen Baby Heart Study

**DOI:** 10.1186/s12916-023-03207-9

**Published:** 2023-12-18

**Authors:** Mette Marie Olsen Nørregaard, Saima Basit, Anne-Sophie Sillesen, Anna Axelsson Raja, Finn Stener Jørgensen, Kasper Karmark Iversen, Henning Bundgaard, Heather Allison Boyd, Ruth Ottilia Birgitta Vøgg

**Affiliations:** 1 Department of Cardiology, Copenhagen University Hospital Herlev-Gentofte, Herlev, Denmark; 2 Department of Clinical Medicine, University of Copenhagen, Copenhagen, Denmark; 3 Department of Epidemiology, Statens Serum Institut, Copenhagen, Denmark; 4 Department of Cardiology, The Heart Centre, Copenhagen University Hospital Rigshospitalet, Copenhagen, Denmark; 5 Fetal Medicine Unit, Department of Obstetrics and Gynecology, Copenhagen University Hospital Hvidovre, Hvidovre, Denmark; 6 Unit for Inherited Cardiac Diseases, Department of Cardiology, The Heart Centre, Copenhagen University Hospital Rigshospitalet, Copenhagen, Denmark

**Keywords:** Newborn heart, Maternal factors, Body mass index, Maternal age, Echocardiography

## Abstract

**Background:**

Maternal obesity and advanced age have been associated with an increased risk of structural congenital heart defects in the offspring. Whether these factors may also cause abnormalities in infant cardiac dimension and function is unknown. This study investigates whether maternal body mass index (BMI) and maternal age are associated with changes in left ventricular (LV) dimensions and function in the newborn.

**Methods:**

Infants enrolled in the Copenhagen Baby Heart Study (CBHS), who were born at term, and contributed with a transthoracic echocardiography (TTE) within 60 days of birth were included. The exposure variables were prepregnancy maternal BMI (kg/m^2^) < 18.5; 18.5–24.9 (reference); 25–29.9; 30–34.9 and ≥ 35 and maternal age (years) < 25; 25–29; 30–34 (reference); 35–39 and ≥ 40. Outcomes were LV parameters ascertained by 2D-echocardiography. Associations between each maternal factor and infant LV parameters were analysed with either a linear model adjusted for the child’s weight and length at birth, gestational age, sex, age at TTE, and maternal smoking, or a linear mixed model, further adjusted for random effects of analyst and month of analysis. Analyses investigating impact of maternal BMI were adjusted for maternal age, and vice versa.

**Results:**

The study cohort included 24,294 infants. Compared with infants in the BMI reference group, infants born to women with a BMI ≥ 25 kg/m^2^ generally had smaller measures of LV internal diameters in end-diastole, reaching statistical significance for BMI 30–34.9 kg/m^2^ [-0.11 ± 0.04 mm, *p* = 0.01]. All groups of infants born to women with a BMI ≥ 25 kg/m^2^ had significantly smaller LV internal diameters in end-systole: BMI 25–29.9 kg/m^2^ [-0.04 ± 0.02 mm, *p* = 0.04], BMI 30–34.9 kg/m^2^ [-0.12 ± 0.03 mm, *p* = 0.001] and BMI ≥ 35 kg/m^2^ [-0.11 ± 0.05 mm, *p* = 0.03].

Compared with infants in the age reference group, infants born to women ≥ 40 years had significantly smaller LV internal diameters in end-diastole [-0.15 ± 0.04 mm, *p* = 0.001] and end-systole [-0.09 ± 0.04 mm, *p* = 0.009].

**Conclusions:**

Systematic population-based echocardiography of infants showed that a maternal prepregnancy BMI ≥ 25 kg/m^2^ and maternal age ≥ 40 years were associated with smaller systolic and diastolic LV diameters. The long-term effects are unknown.

**Clinical trial registration:**

April 2016, Copenhagen Baby Heart, NCT02753348.

**Supplementary Information:**

The online version contains supplementary material available at 10.1186/s12916-023-03207-9.

## Background

Congenital heart defects (CHD) are the most common congenital defects, ranging from complex defects associated with substantial morbidity and mortality to milder forms with less severe physiological impact [1–3]. Our understanding of the factors that influence cardiac development and malformation is incomplete, but the majority of CHD cannot be explained by a single factor and most likely involves a complex interplay of genetic predispositions and environmental risk factors, the latter including maternal risk factors [1, 4, 5].

Maternal body mass index (BMI) above the normal range has been associated with offspring CHD overall [5–10] and with specific cardiac defects [5, 7–11], with some studies reporting increasing odds ratios for infant CHD with increasing degree of maternal obesity [8–10]. In addition, increased risk of atrial septal defects has been reported for women with low (< 18.5) prepregnancy BMI [12]. Maternal age ≥ 35 years [5, 13–15] or ≥ 40 years [5, 16] has also been associated with offspring CHD, both overall [5, 15, 16] and for specific defects [5, 13–15].

Major CHD arise due to errors in cardiac development, which is completed by week eight of gestation. Whether continuous exposure to maternal risk factors during pregnancy may also affect the fetal heart after its formation, leading to subtle structural and functional changes, is unclear.

To date, no large population-based study has investigated the degree to which maternal factors are associated with offspring cardiac structure and function beyond classical CHD. We used data from the Copenhagen Baby Heart Study to assess associations between maternal BMI and age and newborn left ventricular (LV) structural and functional characteristics.

## Methods

### The Copenhagen Baby Heart Study (CBHS)

The CBHS is a population-based cohort study of newborn and childhood cardiac health [17, 18]. From April 2016 to October 2018, all expectant parents from Rigshospitalet, Hvidovre Hospital, and Herlev Hospital (the three largest tertiary centres with maternity wards in the capital region of Denmark) were invited to have their newborns participate in the CBHS. The newborns were examined using systematic transthoracic echocardiography (TTE) within 60 days of delivery [17, 18].

### Study cohort

The study cohort included all newborns in the CBHS who were born at term (≥ 37 gestational weeks) and who contributed with a TTE examination of sufficient quality to allow for analysis. Infants born prematurely (< 37 weeks) were excluded from the study, as were infants with fewer than three core parameters (parameters measured directly from the echocardiogram, rather than calculated based on other measured parameters) measurable by TTE.

### Exposures

Data on maternal age at delivery, maternal height and pre-pregnancy weight were obtained from the Danish Fetal Medicine Database, the Obstetrical Database common to the three participating hospitals [19, 20], and electronic health records. Data, including prenatal screening results, have been collected in the Danish Fetal Medicine Database at all obstetrics departments in Denmark since January 1, 2008 [19]. The Obstetrical Database contains data on all deliveries at Copenhagen University Hospital Rigshospitalet, Hvidovre Hospital and Herlev Hospital, including private birth clinics and home births in the areas covered by these hospitals [20]. From May 2016 manual registration in the Obstetrical database was discontinued at Herlev Hospital. In order to ensure data supply from this hospital, the CBHS collected patient data directly from electronic health records and the Danish National Patient Register [21]. Maternal age was calculated as maternal birth date minus infant birth date rounded down to integers.

Maternal BMI ranges were categorized according to The World Health Organization’s definitions [22] as underweight (< 18.5 kg/m^2^), normal (18.5–24.9 kg/m^2^, reference group), pre-obese (25–29.9 kg/m^2^), obesity class I (30–34.9 kg/m^2^), and obesity classes II + III (≥ 35 kg/m^2^). Maternal age intervals were categorized as < 25 years, 25–29 years, 30–34 years (reference group), 35–39 years, and ≥ 40 years.

### Outcomes

The CBHS’ standard TTE protocol included sub-xiphoid, apical, left parasternal and suprasternal views acquired with cardiac sector transducers 12S-D and 6S-D using Vivid E9 ultrasound equipment (General Electric, Horten, Norway) [17]. The following LV parameters were ascertained from two-dimensional echocardiography in the parasternal long-axis (PLAX) view: interventricular septal end-diastolic thickness (IVSd), LV posterior wall end-diastolic thickness (LVPWd), LV internal diameter in end-diastole (LVIDd), LV internal diameter in end-systole (LVIDs). End-diastolic and end-systolic volume (EDV and ESV), fractional shortening (FS), ejection fraction (EF) and stroke volume (SV) were calculated using Teichholz’ formulae by the General Electric Vivid 9 system [23]. The following diastolic parameters were ascertained in the apical four-chamber view using pulsed-wave Doppler: peak flow velocities across the mitral valve during early diastole (MvE) and atrial contraction (MvA), along with mitral valve E wave deceleration time (MvDT). Heart rate was measured from the 5-chamber view using pulsed-wave Doppler in the LV outflow tract. All measurements were performed in accordance with the standards of the Paediatric Council of the American Society of Echocardiography [24]. To ensure quality and accuracy of LV structural measurements, a team of highly trained and closely supervised project staff (16 people) validated all two-dimensional LV echocardiographic measurements from PLAX-images for the entire CBHS cohort. If the members of this team had any doubts regarding measurements or image quality, the measurements were reviewed by a senior expert in echocardiography. Additionally, all FS measurements ≤ 28% were reviewed by an expert in echocardiography.

### Covariates

Information on newborn sex, gestational age at birth, birth weight, birth length and maternal self-reported smoking status in the first trimester was obtained from the Obstetrical Database [20], the Danish Fetal Medicine Database [19], the local Astraia Fetal Medicine Database and electronic health records.

### Statistical analyses

Association between each maternal factor and newborn structural cardiac parameters measured from PLAX images, as well as functional parameters calculated from these measurements, were assessed using a linear mixed model. Association between maternal factors and newborn heart rate as well as diastolic parameters were assessed using a linear model.

All analyses were adjusted for newborn sex and maternal gestational smoking as categorical variables, gestational age at birth (days), birth length (cm), birth weight (grams), and age at TTE (days) as continuous variables, an approach that has previously been used by the CBHS to accommodate the differences in cardiac dimensions associated with infant size and age [25]. Furthermore, the analyses investigating the impact of maternal prepregnancy BMI were adjusted for maternal age and vice versa. The linear mixed analyses were further adjusted for potential inter- and intra-analyst variability in measuring LV parameters by including random effects for analyst and month within analyst in our models. To remove unrealistic outliers in outcome measurements, all measurements > 10 standard deviations from the mean were removed, and subsequently all measurements > 5 standard deviations from the recalculated mean were also removed from all analyses.

### Sensitivity analyses

To test the robustness of observed associations, we performed sensitivity analyses excluding infants exposed to maternal hypertensive disorders of pregnancy and infants with CHD identified by the CBHS, see additional material for specification. In addition, we repeated the maternal BMI analyses excluding children born to mothers with pregestational or gestational diabetes.

## Results

**Fig. 1:**
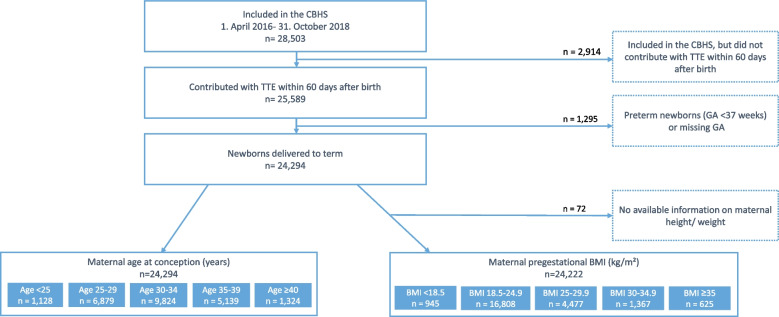
Flowchart illustrating the construction of the study cohort

Of the 25,589 CBHS infants with a valid TTE examination, 24,294 were born at term (≥ 37 weeks) and were included in the study cohort (Fig. 1). Characteristics of the cohort are presented in Tables 1 and 2. More than 40% of children were born to mothers in their early thirties, whilst 5.4% were born by mothers ≥ 40 years of age. The majority of children were born to mothers with a prepregnancy BMI between 18.5–24.9 with just 5.6% born to women with a BMI between 30–34.9 and 2.6% to mothers with a BMI ≥ 35.

**Table 1:** Maternal and newborn characteristics by maternal body mass index in a cohort of 24,222 infants from the Copenhagen Baby Heart Study, Denmark, 2016–2018

	Number (%)				
Maternal body mass index	Underweight BMI < 18.5 *N* = 945	Normal BMI 18.5–24.9 *N* = 16808	Preobesity BMI 25.0–29.9 *N* = 4477	Obesity class I BMI 30.0–34.9 *N* = 1367	Obesity class II + III BMI ≥ 35 *N* = 625
Maternal Characteristics					
Age at delivery (years)					
< 25	71 (7.5)	711 (4.2)	216 (4.8)	78 (5.7)	41 (6.6)
25–29	296 (31.3)	4740 (28.2)	1261 (28.2)	377 (27.6)	188 (30.1)
30–34	388 (41.1)	6961 (41.4)	1721(38.4)	506 (37.0)	222 (35.5)
35–39	160 (16.9)	3520 (20.9)	982 (21.9)	323 (23.6)	141 (22.6)
≥ 40	30 (3.2)	876 (5.2)	297 (6.6)	83 (6.1)	33 (5.3)
First-trimester smoking					
Yes	40 (4.2)	405 (2.4)	157 (3.5)	92 (6.7)	45 (7.2)
No	870 (92.1)	15985 (95.1)	4185 (93.5)	1254 (91.7)	555 (88.8)
Missing	35 (3.7)	418 (2.5)	135 (3.0)	21 (1.5)	25 (4.0)
Newborn Characteristics					
Sex					
Male	500 (52.9)	8620 (51.3)	2329 (52.0)	750 (54.9)	308 (49.3)
Female	445 (47.1)	8188 (48.7)	2148 (48.0)	617 (45.1)	317 (50.7)
Gestational age at birth (weeks)					
37	61 (6.5)	915 (5.4)	281 (6.3)	110 (8.0)	74 (11.8)
38	148 (15.7)	2136 (12.7)	648 (14.5)	238 (17.4)	132 (21.1)
39	234 (24.8)	3681 (21.9)	925 (20.7)	298 (21.8)	118 (18.9)
40	296 (31.3)	5275 (31.4)	1260 (28.1)	367 (26.8)	135 (21.6)
≥ 41	206 (21.8)	4801 (28.6)	1363 (30.4)	354 (25.9)	166 (26.6)
Birthweight (g)					
< 2500	^*^	264 (1.6)	46 (1.0)	^*^	10 (1.6)
2500–2999	172 (18.2)	1934 (11.5)	402 (9.0)	126 (9.2)	57 (9.1)
3000–3499	380 (40.2)	6014 (35.8)	1397 (31.2)	414 (30.3)	166 (26.6)
3500–3999	301 (31.9)	6184 (36.8)	1676 (37.4)	482 (35.3)	237 (37.9)
≥ 4000	66 (7.0)	2389 (14.2)	949 (21.2)	313 (22.9)	155 (24.8)
Missing	^*^	23 (0.1)	7 (0.2)	^*^	0 (0.0)
Birth length (cm)					
< 50	167 (17.7)	2177 (13.0)	486 (10.9)	^*^	^*^
50–54.9	717 (75.9)	13031 (77.5)	3436 (76.7)	1046 (76.5)	468 (74.9)
≥ 55	54 (5.7)	1531 (9.1)	533 (11.9)	143 (10.5)	70 (11.2)
Missing	7 (0.7)	69 (0.4)	22 (0.5)	^*^	^*^
Congenital heart defect^a^					
Yes	66 (7.0)	1126 (6.7)	339 (7.6)	116 (8.5)	45 (7.2)
No	879 (93.0)	15682 (93.3)	4138 (92.4)	1251 (91.5)	580 (92.8)

72 infants had mothers who were missing information regarding BMI

^*^Data not shown in order to avoid compromising personal data

^a^Any of the following defects found by the CBHS by use of postnatal TTE: Atrial septal defects, ventricular septal defects, bicuspid aortic valve, quadricuspid aortic valve, aortic stenosis, pulmonic stenosis, quadricuspid pulmonic valve, coarctation of the aorta, tetraology of Fallot, transposition of the great arteries, atrioventricular septal defects, congenitally corrected transposition of the great arteries, situs inversus, Epsteins anomaly, cardiogenic tumors and supradiaphragmatic totally anomalous pulmonary venous return

**Table 2:** Maternal and newborn characteristics by maternal age at delivery in a cohort of 24,294 infants from the Copenhagen Baby Heart Study, Denmark, 2016–2018

Maternal age at delivery	Number (%)				
	< 25 years *N* = 1128	25–29 years *N* = 6879	30–34 years *N* = 9824	35–39 years *N* = 5139	≥ 40 years *N* = 1324
Maternal Characteristics					
Maternal prepregnancy BMI					
< 18.5	71 (6.3)	296 (4.3)	388 (3.9)	160 (3.1)	30 (2.3)
18.5–24.9	711 (63.0)	4740 (68.9)	6961 (70.9)	3520 (68.5)	876 (66.2)
25.0–29.9	216 (19.1)	1261 (18.3)	1721 (17.5)	982 (19.1)	297 (22.4)
30.0–34.9	78 (6.9)	377 (5.5)	506 (5.2)	323 (6.3)	83 (6.3)
≥ 35	41 (3.6)	188 (2.7)	222 (2.3)	141 (2.7)	33 (2.5)
Missing	11 (1.0)	17 (0.2)	26 (0.3)	13 (0.3)	5 (0.4)
First-trimester smoking					
Yes	138 (12.2)	220 (3.2)	234 (2.4)	119 (2.3)	29 (2.2)
No	934 (82.8)	6480 (94.2)	9343 (95.1)	4867 (94.7)	1260 (95.2)
Missing	56 (5.0)	179 (2.6)	247 (2.5)	153 (3.0)	35 (2.6)
Newborn Characteristics					
Sex					
Male	607 (53.8)	3498 (50.9)	5053 (51.4)	2704 (52.6)	683 (51.6)
Female	521 (46.2)	3381 (49.1)	4771 (48.6)	2435 (47.4)	641 (48.4)
Gestational age at birth (weeks)					
37	84 (7.4)	377 (5.5)	537 (5.5)	322 (6.3)	121 (9.1)
38	143 (12.7)	889 (12.9)	1255 (12.8)	783 (15.2)	245 (18.5)
39	266 (23.6)	1529 (22.2)	2127 (21.7)	1062 (20.7)	288 (21.8)
40	348 (30.9)	2134 (31.0)	2970 (30.2)	1530 (29.8)	373 (28.2)
≥ 41	287 (25.4)	1950(28.3)	2935 (29.8)	1442 (28.0)	297(22.4)
Birthweight (g)					
< 2500	^*^	92 (1.3)	128 (1.3)	90 (1.8)	^*^
2500–2999	140 (12.4)	713 (10.4)	1115 (11.3)	540 (10.5)	193 (14.6)
3000–3499	406 (36.0)	2424 (35.2)	3390 (34.5)	1733 (33.7)	439 (33.2)
3500–3999	389 (34.5)	2620 (38.1)	3596 (36.6)	1856 (36.1)	444 (33.5)
≥ 4000	167 (14.8)	1018 (14.8)	1580 (16.1)	914 (17.8)	204 (15.4)
Missing	^*^	12 (0.2)	15 (0.2)	6 (0.1)	^*^
Birth length (cm)					
< 50	174 (15.4)	828 (12.0)	1236 (12.6)	638 (12.4)	225 (17.0)
50–54.9	860 (76.2)	5396 (78.4)	7555 (76.9)	3964 (77.1)	969 (73.2)
≥ 55	87 (7.7)	624 (9.1)	990 (10.1)	517 (10.1)	123 (9.3)
Missing	7 (0.6)	31 (0.5)	43 (0.4)	20 (0.4)	7 (0.5)
Congenital heart defect^a^					
Yes	74 (6.6)	478 (6.9)	708 (7.2)	346 (6.7)	92 (6.9)
No	1054 (93.4)	6401 (93.1)	9116 (92.8)	4793 (93.3)	1232 (93.1)

^*^Data not shown in order to avoid compromising personal data

^a^Any of the following defects found by the CBHS by use of postnatal TTE: Atrial septal defects, ventricular septal defects, bicuspid aortic valve, quadricuspid aortic valve, aortic stenosis, pulmonic stenosis, quadricuspid pulmonic valve, coarctation of the aorta, tetraology of Fallot, transposition of the great arteries, atrioventricular septal defects, congenitally corrected transposition of the great arteries, situs inversus, Epsteins anomaly, cardiogenic tumors and supradiaphragmatic totally anomalous pulmonary venous return

**Fig. 2:**
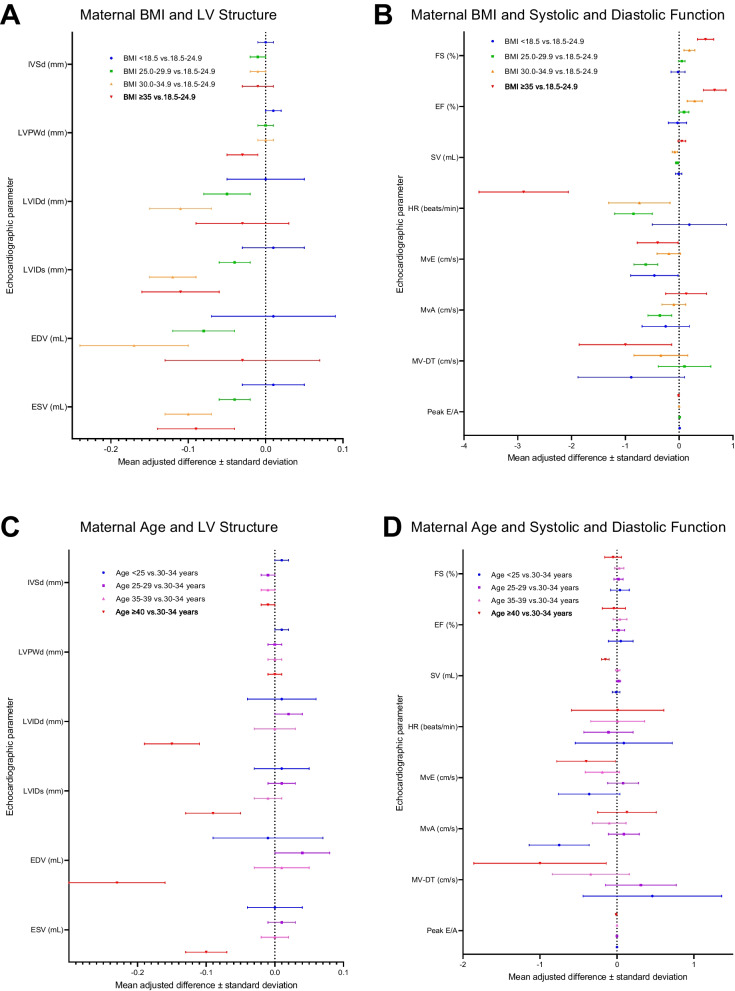
Associations between maternal characteristics and offspring left ventricular parameters. Panels A and B show associations between maternal BMI and offspring left ventricular structure (**A**) and systolic and diastolic function (**B**), where children born to women with BMIs in the following groups were compared with children born to women with BMI 18.5–24.9 (reference group): blue, BMI < 18.5; green, BMI 25.0–29.9; orange, BMI 30.0–34.9; red, BMI ≥ 35.0. Panels C and D show associations between maternal age and offspring left ventricular structure (**C**) and systolic and diastolic function (**D**), where children born to women in the following age groups were compared with children born to women aged 30–34 years (reference group): blue, <25 years; purple, 25–29 years; pink, 35–39 years; purple, ≥ 40 years

Associations between maternal BMI and newborn LV parameters are presented in Fig. 2, panels A and B (Additional file 1: Table S1). Infants born to women with a prepregnancy BMI ≥ 25 generally had smaller dimensions of the ventricular cavity (LVIDd, LVIDs, EDV and ESV) than infants born to women with a prepregnancy BMI in the normal range (adjusted mean difference [aMD] ± standard deviation [SD] for infants born to pre-obese women: LVIDd, -0.05 ± 0.03 mm, *p* = 0.06; LVIDs, -0.04 ± 0.02 mm, *p* = 0.04; EDV, -0.08 ± 0.04 ml, *p* = 0.06; ESV, -0.04 ± 0.02 ml, *p* = 0.06; aMD ± SD for infants born to women in obesity class I: LVIDd, -0.11 ± 0.04 mm, *p* = 0.01; LVIDs, -0.12 ± 0.03 mm, *p* = 0.001; EDV, -0.17 ± 0.07 ml, *p* = 0.01; ESV, -0.10 ± 0.03 ml, *p* = 0.002; aMD ± SD for infants born to women in obesity classes II or III: LVIDd, -0.03 ± 0.06 mm, *p* = 0.60; LVIDs, -0.11 ± 0.05 mm, *p* = 0.03; EDV, -0.03 ± 0.10 ml, *p* = 0.73; ESV, -0.09 ± 0.05 ml, *p* = 0.04). In contrast, we saw no pattern of association between maternal BMI and the thickness of the ventricular walls (IVSd and LVPWd) of the newborns (Fig. 2, panel A; Additional file 1: Table S1).

A maternal BMI ≥ 30 was associated with increased systolic function (FS and EF) in the offspring (aMD ± SD for infants born to women in obesity class I: FS, 0.19 ± 0.10%, *p* = 0.06; EF, 0.29 ± 0.14%; *p* = 0.04, aMD ± SD for infants born to women in obesity classes II or III: FS, 0.49 ± 0.15%, *p* = 0.002; EF, 0.66 ± 0.21%, *p* = 0.002). Infants born to women with a BMI in the preobese or obesity class II/III ranges had decreased heart rates (-0.85 ± 0.35 beats/min, *p* = 0.01 and -2.89 ± 0.83 beats/min, *p* = 0.001, respectively), compared with infants born to women whose BMI was in the normal range. Generally, infants born to women with a BMI ≥ 25 had smaller measures of MvE and MvA (aMD ± SD for infants born to pre-obese women: MvE. -0.62 ± 0.22 cm/sec, *p* = 0.004; MvA, -0.36 ± 0.22 cm/sec, *p* = 0.1; aMD ± SD for infants born to women in obesity class I: MvE, -0.57 ± 0.36 cm/sec, *p* = 0.11; MvA, -0.24 ± 0.36 cm/sec, *p* = 0.51; aMD ± SD for infants born to women in obesity classes II or III: MvE, -0.91 ± 0.52 cm/sec *p* = 0.08; MvA, -1.02 ± 0.53 cm/sec, *p* = 0.05) but not with other measures of diastolic function (Fig. 2, panel B; Additional file 1: Table S1). We found no evidence of association between maternal underweight and any infant LV parameter (Fig. 2, panels A and B; Additional file 1: Table S1).

Panels C and D of Fig. 2 show associations between maternal age and newborn LV parameters (Additional file 1: Table S2). Compared with children born to mothers 30–34 years of age, children born to women ≥ 40 years of age had smaller  LV cavity dimensions, aMD± SD: LVIDd, -0.15 ± 0.04 mm, *p* = 0.001; LVIDs, -0.09 ± 0.04 mm, *p* = 0.009; EDV, -0.23 ± 0.07 ml, *p* = 0.001; ESV, -0.1 ± 0.03 ml, *p* = 0.004; SV, -0.15 ± 0.05 ml, *p* = 0.002. There was no evidence of an association between maternal age ≥ 40 years and other cardiac parameters investigated in this study, nor did we observe associations between any other maternal age group and any infant LV parameter (Fig. 2, panels C and D; Additional file 1: Table S2).

Excluding infants born to women with hypertensive disorders of pregnancy increased the significance of association with smaller LVIDd, EDV and ESV for the pre-obese group, but did not change the overall pattern of association between maternal BMI and infant LV parameters (Additional file 1: Table S3). For the analysis investigating the association between maternal age at delivery and infant LV parameters, excluding women with hypertensive disorders of pregnancy did not meaningfully change the associations (Additional file 1: Table S4).

Excluding infants with CHD attenuated some of the associations with pre-obesity and obesity, although the general pattern of association remained (Additional file 1: Table S5). However, excluding infants with CHD did not alter the associations for maternal age (Additional file 1: Table S6). Excluding infants born to mothers with pregestational or gestational diabetes did not change the overall pattern of association between maternal BMI and infant LV parameters (Additional file 1: Table S7).

## Discussion

The results of our large population-based cohort study suggest that maternal BMI above the normal range is associated with altered LV geometry and lower LV volume in the newborn heart. Maternal obesity (class I + II + III) were also associated with changes in systolic function, including increased FS and EF whilst pre-obesity and obesity class II + III were associated with decreased heart rate. In contrast, among measures of diastolic function, maternal pre-obesity and obesity were only associated with decreased MvA. Infants born to mothers ≥ 40 years of age had smaller LV internal diameters and decreased stroke volume compared to infants born to mothers in their early thirties; otherwise, there were no associations between maternal age and newborn LV parameters.

Previous echocardiographic studies that assessed associations between maternal BMI and infant cardiac parameters differed from our study in terms of cohort size, the age of study participants, the definition of maternal BMI ranges used for exposure, and outcome measurements [26–29]. Guzzardi et al. (*N* = 30) found no significant differences in LV mass or LVPWd in newborns to overweight women compared with newborns to women with normal pre-pregnancy BMIs [27], and Geelhoed et al. (*N* = 791) found no association between maternal BMI and infant LV mass at 6 weeks and 6 months of age [26]. Litwin et al. (*N* = 201) found no association between maternal pre-pregnancy BMI and LV mass in offspring at age 6 years [28]. Groves et al. found no difference in LV mass assessed using cardiac MRI between neonates < 72 h of age born to women with BMI ≥ 30 (*N* = 13) and neonates born to women with BMI 20–25 (*N* = 20) [29]. These findings are consistent with our findings of no association between maternal BMI and newborn LV wall thickness.

In their comparison of newborns to overweight and normal weight mothers, Guzzardi et al. found no significant difference in EDV, ESV, SV or EF [27]. In contrast, Groves et al. showed that EDV and SV were decreased in very young neonates born to obese women [29], which is more consistent with our finding of an association between maternal pre-obesity and obesity and lower offspring LV volumes. Although we found no significant association with SV, we did observe an association between obesity (class I + II + II) and increased EF.

In contrast to our finding of lower heart rates in infants of pre-obese and obese mothers, Groves et al. found higher heart rates ≤ 72 h after birth in infants born to obese women [29]. The ages of the infants in our study are between a few hours to 60 days old which is a possible explanation for the difference in findings. Another possible explanation for this difference is that Groves et al. attained the heart rate as an overall average of a 25-min electrocardiogram, which was further stratified by sleep or wakefulness of the newborn, whilst our study measured heart rate in a single echocardiographic loop a few seconds in length unstratified by the activity level of the child. The heart rate of a newborn can vary greatly within a few seconds, and this variability is not accounted for when assessing heart rate from a short echocardiographic loop rather than an average of a longer sequence.

To our knowledge, no study has investigated the association between maternal age and LV echocardiographic parameters in the newborn, although there is evidence that advanced maternal age is associated with overt CHD [5, 13, 14]. We found that maternal age ≥ 40 years of age was associated with smaller values of infant LV cavity dimensions and SV, compared with maternal age between 30 and 34 years. Excluding infants with CHD from the analyses changes our results very little, indicating that our observations did not simply reflect an association between advanced maternal age and CHD.

Hypertensive disorders of pregnancy, including preeclampsia, affect up to 10% of pregnancies [30–32] and are associated with both increased maternal BMI and advanced maternal age [33]. Preeclampsia is also associated with offspring CHD [34–36]. Excluding infants born to women with hypertensive disorders of pregnancy from the analyses investigating impact of maternal BMI increased significance of association smaller measures of LVIDd, EDV, ESV and maternal BMI in the preobese range. A previous CBHS study found an association between preeclampsia and increased measures of newborn LVIDd and EDV,^17^ which may explain why removing this group of infants strengthens association between BMI and smaller LVIDd and EDV measurements in the newborn. Excluding infants born to women with hypertensive disorders of pregnancy did not change the associations between maternal age and infant LV parameters, suggesting that hypertensive disorders of pregnancy cannot explain our findings.

Maternal diabetes has been suggested as an explanation for associations between maternal obesity and offspring CHD [6]. Excluding infants born to mothers with pregestational or gestational diabetes did not meaningfully alter the found association with newborn LV parameters. Excluding infants with CHD attenuated the observed associations somewhat, suggesting that known associations between obesity, diabetes, and CHD could account for some, but not all, of our findings.

The between-group differences in LV parameters that we report in this study are small in absolute terms. However, the newborn heart is tiny and a 0.1 mm decrease in LVIDs represents an 0.8% decrease in LV internal diameter. Infants in the study were asymptomatic, despite these subtle changes, and consequently not affected clinically by our findings. Whether these subtle differences in cardiac parameters observed shortly after birth have long-term clinical significance is currently unknown, and follow-up studies are required to determine whether these differences persist or worsen and therefore whether fetal exposure to maternal factors has long-term consequences for offspring cardiac health. If it does, maternal overweight and obesity and advanced maternal age, may come to explain an increasing proportion of the population burden of cardiovascular disease. Both the proportion of expecting mothers with an above-normal BMI and the average age at first birth in Denmark are increasing [37, 38].

### Strengths and limitations

A major strength of our study was the size of the study population, which allowed us power to detect between-group differences in cardiac parameters that might have gone unnoticed in smaller cohorts, to analyse fine categories of BMI and age and add nuance to previous findings, and to conduct extensive sensitivity analyses to ensure that observed associations were not being driven by subgroups with underlying illnesses. The CBHS’ birth cohort design, population-based nature and minimal exclusions minimised the risk of selection bias. Both the echocardiographers and project staff who validated the LV measurements from PLAX-images were blinded to maternal pre-pregnancy BMI and age at delivery minimizing the chance of ascertainment bias. Finally, we make an important contribution to the literature by demonstrating the usefulness of echocardiography with a standardized protocol tailored to the newborn in addressing important questions in paediatric cardiology. While we acknowledge cardiac MRI, as implemented by Groves et al. [26], as the gold standard, TTE is less expensive, easier to implement in a wide variety of settings, and generally results in excellent image quality in neonates.

The CBHS’ echocardiographic data showed good intra- and interobserver agreement for most two-dimensional LV parameters (interclass correlation coefficients [ICC] ≥ 0.67), with the exception of interobserver agreement for LVPWd (ICC = 0.15) and IVSd (ICC = 0.19) measurements [39]. To minimize variability in structural LV parameters, all LV parameters measured in the PLAX view were validated by a team of 16 project employees. Furthermore, to account for any remaining inter- and intra-analyst variability, we adjusted for random effects of analyst and month of analysis in the linear mixed model.

Because the circulatory system undergoes massive physiological changes in the weeks after birth, and cardiac structures increase rapidly in size commensurate with rapid somatic growth in infants, cardiac dimensions and other parameters in infants are strongly correlated with body size and age [40–42]. To avoid potential biases arising from comparing raw measurements from infants scanned across a 60-day period, we adjusted our results for infant birth length and birthweight, gestational age at birth, and age at TTE, which we have shown in previous work accounts well for age- and size-related differences in infant LV parameters measured using TTE [25]. Furthermore, although infants could theoretically be up to 60 days of age at TTE, 96% were examined within 4 weeks of birth, minimizing the impact of the large age spread. While we could have limited our study population to children examined within the first 4 weeks of life, doing so would have excluded a disproportionate number of babies who spent time in the neonatal unit in the first weeks of life and we felt it was important to include as many such children as possible.

Although all children born at participating hospitals in the study period were eligible to participate in the CBHS, infants born to underweight, overweight or obese women, women < 25 years of age, or smokers were less likely to participate than infants born to women with normal BMI, women > 25 years of age, and non-smokers [18]. In particular, there were too few infants born to women in obesity classes II and III to analyse them in separate categories, and even when we aggregated the groups, we lacked power to draw firm conclusions about these children.

Although all expectant parents delivering at participating hospitals were invited to participate in the CBHS, children from higher-income households, those with well-educated mothers and those of Danish origin were overrepresented in the cohort. Furthermore, the three participating hospitals serve an exclusively urban population. While differences between CBHS participants and non-participants in the distributions of socioeconomic and demographic factors should not have affected the validity of our findings, they may not be generalizable to other populations.

Excessive, long-term maternal alcohol use during pregnancy is known to be associated with CHDs. Lesser levels of maternal alcohol consumption during the periconceptional period and pregnancy have previously also been linked to CHDs in the offspring, although the strength of the association varied according to the composition of the study population and the definition of alcohol consumption [43–45]. Ideally, therefore, we would have adjusted our results for maternal alcohol use. However, information on maternal alcohol consumption is not registered systematically in the electronic medical records in Denmark. Data on maternal alcohol use were only available for approximately 10% of our cohort, making it impossible to impute maternal alcohol use patterns where this information was missing, particularly since only a handful of mothers reported any drinking during pregnancy. Consequently, we were unable to adjust our findings for maternal alcohol use. Levels of drinking among pregnant women have been reported to vary with maternal age [46], with older pregnant women (> 34 years old) 37% more likely to report any drinking during pregnancy than younger women; however, self-reported rates of binge drinking did not differ significantly for young (18–24 years) and older (> 34 years old) pregnant women (2.5% vs. 1.8%). This suggests, fortunately, that occasional, light maternal alcohol use (the type expected in our study population) is unlikely to have been a strong confounder, at least of the associations between maternal age and infant LV parameters.

Our study used self-reported maternal height and pre-pregnancy weight. Generally, adults tend to underestimate their weight and overestimate their height [47, 48]. However, Seijo et al. found that self-reported data on height and weight by women of reproductive age closely estimated actual values, leading them to conclude that such data can be a useful proxy for measured values [49].

## Conclusions

Maternal BMI above the normal range and maternal age ≥ 40 years were associated with changes in LV cavity dimensions and functional parameters in the offspring. Thus, our results suggest that these maternal risk factors are not only associated with CHD but may also affect offspring LV structure and function in more subtle ways.

### Supplementary Information

**Additional file 1: Table S1.** Adjusted mean differences comparing left ventricular parameters in infants born to underweight, pre-obese or obese women and in infants born to women with normal pre-pregnancy BMIs, in a cohort of 24,222 infants from the Copenhagen Baby Heart Study, 2016-2018. **Table S2.** Adjusted mean differences comparing left ventricular parameters in infants born to women aged <25 years, 25-29 years, 35-39 years and ≥40 years with those in infants born to women with aged 30-34 years, in a cohort of 24,294 infants from the Copenhagen Baby Heart Study, 2016-2018. **Table S3.** Adjusted mean differences comparing left ventricular parameters in infants born to underweight, pre-obese or obese women and in infants born to women with normal pre-pregnancy BMIs, excluding from the cohort infants born to women with gestational hypertension or preeclampsia. **Table S4.** Adjusted mean differences comparing left ventricular parameters in infants born to women aged <25 years, 25-29 years, 35-39 years and ≥40 years with those in infants born to women with aged 30-34 years, excluding from the cohort infants born to women with gestational hypertension or preeclampsia. **Table S5.** Adjusted mean differences comparing left ventricular parameters in infants born to underweight, pre-obese or obese women and in infants born to women with normal pre-pregnancy BMIs, excluding from the cohort infants in whom the Copenhagen Baby Heart Study identified a congenital heart defect. **Table S6.** Adjusted mean differences comparing left ventricular parameters in infants born to women aged <25 years, 25-29 years, 35-39 years and ≥40 years with those in infants born to women with aged 30-34 years, excluding from the cohort infants in whom the Copenhagen Baby Heart Study identified a congenital heart defect. **Table S7.** Adjusted mean differences comparing left ventricular parameters in infants born to underweight, pre-obese or obese women and in infants born to women with normal pre-pregnancy BMIs, excluding from the cohort infants born to women with pre-gestational or gestational diabetes.

## Data Availability

The data used in the article cannot be shared publicly due to Danish and EU data protection laws. However, the data may be shared for specific, well-defined projects that are approved by the Danish Data Protection Agency, the Scientific Ethics Committees of the Capital City Region of Denmark, and the CBHS’ Steering Committee.

## Abbreviations

aMD: Adjusted Mean Difference
BMI: Body Mass Index
CBHS: Copenhagen Baby Heart Study
CHD: Congenital Heart Defects
EDV: End-Diastolic Volume
EF: Ejection Fraction
ESV: End-Systolic Volume
FS: Fractional Shortening
IVSd: Interventricular Septal end-Diastolic thickness
LV: Left Ventricle
LVIDd: Left Ventricular Internal Diameter in end-Diastole
LVIDs: Left Ventricular Internal Diameter in end-Systole
LVPWd: Left Ventricular Posterior Wall end-Diastolic thickness
MvA: Peak flow velocity across the Mitral Valve during Atrial contraction
MvE: Peak flow velocity across the Mitral Valve during Early diastole
MvDT: Mitral Valve E wave Deceleration Time
PLAX: Parasternal Long Axis
SD: Standard Deviation
SV: Stroke Volume
TTE: Transthoracic Echocardiography
