# Supplementary material for: Impact of maternal age and body mass index on the structure and function of the heart in newborns: a Copenhagen Baby Heart Study
Source: BMC Med. 2023 Dec 18;21:499. doi: 10.1186/s12916-023-03207-9 (PMC10729451; doi:10.1186/s12916-023-03207-9)
Supplement: Supplementary file 1 — Additional file 1: Table S1. Adjusted mean differences comparing left ventricular parameters in infants born to underweight, pre-obese or obese women and in infants born to women with normal pre-pregnancy BMIs, in a cohort of 24,222 infants from the Copenhagen Baby Heart Study, 2016-2018. Table S2. Adjusted mean differences comparing left ventricular parameters in infants born to women aged <25 years, 25-29 years, 35-39 years and ≥40 years with those in infants born to women with aged 30-34 years, in a cohort of 24,294 infants from the Copenhagen Baby Heart Study, 2016-2018. Table S3. Adjusted mean differences comparing left ventricular parameters in infants born to underweight, pre-obese or obese women and in infants born to women with normal pre-pregnancy BMIs, excluding from the cohort infants born to women with gestational hypertension or preeclampsia. Table S4. Adjusted mean differences comparing left ventricular parameters in infants born to women aged <25 years, 25-29 years, 35-39 years and ≥40 years with those in infants born to women with aged 30-34 years, excluding from the cohort infants born to women with gestational hypertension or preeclampsia. Table S5. Adjusted mean differences comparing left ventricular parameters in infants born to underweight, pre-obese or obese women and in infants born to women with normal pre-pregnancy BMIs, excluding from the cohort infants in whom the Copenhagen Baby Heart Study identified a congenital heart defect. Table S6. Adjusted mean differences comparing left ventricular parameters in infants born to women aged <25 years, 25-29 years, 35-39 years and ≥40 years with those in infants born to women with aged 30-34 years, excluding from the cohort infants in whom the Copenhagen Baby Heart Study identified a congenital heart defect. Table S7. Adjusted mean differences comparing left ventricular parameters in infants born to underweight, pre-obese or obese women and in infants born to women with normal pre-pregnancy BMIs, ex [file 12916_2023_3207_MOESM1_ESM.docx]

**Additional File 1 for “Impact of Maternal Age and Body Mass Index on the Structure and Function of the Heart in Newborns: a Copenhagen Baby Heart Study”**

Table S1. Adjusted mean differences comparing left ventricular parameters in infants born to underweight, pre-obese or obese women with those in infants born to women with normal pre-pregnancy BMIs, in a cohort of 24,222 infants from the Copenhagen Baby Heart Study, 2016-2018.

|  | Mean adjusted difference ± standard deviation (p-value) | | | |
| --- | --- | --- | --- | --- |
|  | Maternal BMI<18.5 (Underweight) | Maternal BMI 25.0-29.9  (Pre-Obese) | Maternal BMI 30.0-34.9  (Obesity class I) | Maternal BMI≥35  (Obesity class II and III) |
| **Left ventricular (LV) structure** |  | | | |
| Interventricular septum in end-diastole, IVSd (mm) | 0.00 ± 0.01 (0.82) | -0.01 ± 0.01 (0.31) | -0.01 ± 0.01 (0.51) | -0.01 ± 0.02 (0.58) |
| LV posterior wall, LVPWd (mm) | 0.01 ± 0.01 (0.58) | 0.00 ± 0.01 (0.77) | 0.00 ± 0.01 (0.78) | -0.03 ± 0.02 (0.10) |
| LV internal diameter in end-diastole, LVIDd (mm) | 0.00 ± 0.05 (0.97) | -0.05 ± 0.03 (0.06) | -0.11 ± 0.04 (0.01) | -0.03 ± 0.06 (0.60) |
| LV internal diameter in end-systole, LVIDs (mm) | 0.01 ± 0.04 (0.90) | -0.04 ± 0.02 (0.04) | -0.12 ± 0.03 (0.001) | -0.11 ± 0.05 (0.03) |
| End-diastolic volume, EDV (ml)^1^ | 0.01 ± 0.08 (0.87) | -0.08 ± 0.04 (0.06) | -0.17 ± 0.07 (0.01) | -0.03 ± 0.10 (0.73) |
| End-systolic volume, ESV (ml)^1^ | 0.01 ± 0.04 (0.70) | -0.04 ± 0.02 (0.06) | -0.10 ± 0.03 (0.002) | -0.09 ± 0.05 (0.04) |
| **Systolic function** |  |  |  |  |
| Fractional shortening, FS (%)^1^ | -0.02 ± 0.13 (0.88) | 0.05 ± 0.06 (0.40) | 0.19 ± 0.10 (0.06) | 0.49 ± 0.15 (0.002) |
| Ejection fraction, EF (%)^1^ | -0.03 ± 0.17 (0.84) | 0.09 ± 0.09 (0.27) | 0.29 ± 0.14 (0.04) | 0.66 ± 0.21 (0.002) |
| Stroke volume, SV (ml)^1^ | -0.01 ± 0.06 (0.87) | -0.04 ± 0.03 (0.14) | -0.08 ± 0.05 (0.09) | 0.05 ± 0.07 (0.44) |
| Heart rate, HR (beats per minute) | 0.19 ± 0.69 (0.78) | -0.85 ± 0.35 (0.01) | -0.74 ± 0.57 (0.20) | -2.89 ± 0.83 (0.001) |
| **Diastolic function** |  | | | |
| Mitral valve early peak velocity, MvE (cm/sec) | -0.46 ± 0.44 (0.30) | -0.62 ± 0.22 (0.004) | -0.57 ± 0.36 (0.11) | -0.91 ± 0.52 (0.08) |
| Mitral valve atrial peak velocity, MvA (cm/sec) | -0.25 ± 0.44 (0.57) | -0.36 ± 0.22 (0.10) | -0.24 ± 0.36 (0.51) | -1.02 ± 0.53 (0.05) |
| Mitral valve deceleration time, MV DT (cm/sec) | -0.89 ± 0.99 (0.37) | 0.10 ± 0.49 (0.84) | 0.45 ± 0.82 (0.58) | 1.65 ± 1.19 (0.16) |
| Peak E/A ratio | 0.01 ± 0.01 (0.52) | 0.01 ± 0.00 (0.08) | 0.01 ± 0.01 (0.32) | 0.01 ± 0.01 (0.62) |
| Adjusted for the child’s gestational age at birth, sex, weight and length at birth, age in days at examination and random effect of echocardiographic analyst and month of analysis.  ^1^Calculated by use of Teichholz’ formula. | | | | |

Table S2. Adjusted mean differences comparing left ventricular parameters in infants born to women aged <25 years, 25-29 years, 35-39 years and ≥40 years with those in infants born to women with aged 30-34 years, in a cohort of 24,294 infants from the Copenhagen Baby Heart Study, 2016-2018.

|  | Mean adjusted difference ± standard deviation (p-value) | | | |
| --- | --- | --- | --- | --- |
|  | Maternal age <25 years | Maternal age 25-29 years | Maternal age 35-39 years | Maternal age ≥40 years |
| **Left ventricular (LV) structure** |  | | | |
| Interventricular septum in end-diastole, IVSd (mm) | 0.01 ± 0.01 (0.58) | -0.01 ± 0.01 (0.04) | -0.01 ± 0.01 (0.31) | -0.01 ± 0.01 (0.42) |
| LV posterior wall, LVPWd (mm) | 0.01 ± 0.01 (0.68) | 0 ± 0.01 (0.78) | 0 ± 0.01 (0.97) | 0 ± 0.01 (0.99) |
| LV internal diameter in end-diastole, LVIDd (mm) | 0.01 ± 0.05 (0.86) | 0.02 ± 0.02 (0.31) | 0 ± 0.03 (0.92) | -0.15 ± 0.04 (0.001) |
| LV internal diameter in end-systole, LVIDs (mm) | 0.01 ± 0.04 (0.77) | 0.01 ± 0.02 (0.47) | -0.01 ± 0.02 (0.81) | -0.09 ± 0.04 (0.009) |
| End-diastolic volume, EDV (ml)^1^ | -0.01 ± 0.08 (0.88) | 0.04 ± 0.04 (0.30) | 0.01 ± 0.04 (0.73) | -0.23 ± 0.07 (0.001) |
| End-systolic volume, ESV (ml)^1^ | 0.00 ± 0.04 (0.98) | 0.01 ± 0.02 (0.50) | 0 ± 0.02 (0.93) | -0.1 ± 0.03 (0.004) |
| **Systolic function** |  |  |  |  |
| Fractional shortening, FS (%)^1^ | 0.04 ± 0.12 (0.99) | 0.02 ± 0.06 (0.77) | 0.03 ± 0.06 (0.62) | -0.05 ± 0.11 (0.62) |
| Ejection fraction, EF (%)^1^ | 0.05 ± 0.16 (0.99) | 0.02 ± 0.08 (0.84) | 0.04 ± 0.09 (0.66) | -0.04 ± 0.15 (0.79) |
| Stroke volume, SV (ml)^1^ | -0.01 ± 0.05 (0.82) | 0.02 ± 0.03 (0.38) | 0.01 ± 0.03 (0.78) | -0.15 ± 0.05 (0.002) |
| Heart rate, HR (beats per minute) | 0.09 ± 0.63 (0.62) | -0.11 ± 0.32 (0.74) | 0.01 ± 0.35 (0.97) | 0.01 ± 0.60 (0.98) |
| **Diastolic function** |  |  |  |  |
| Mitral valve early peak velocity, MvE (cm/sec) | -0.36± 0.40 (0.07) | 0.08± 0.20 (0.69) | -0.19 ± 0.22 (0.39) | -0.40 ± 0.38 (0.29) |
| Mitral valve atrial peak velocity, MvA (cm/sec) | -0.75± 0.39 (0.24) | 0.09 ± 0.20 (0.64) | -0.10 ± 0.22 (0.67) | 0.13 ± 0.38 (0.74) |
| Mitral valve deceleration time, MV DT (cm/sec) | 0.46 ± 0.90 (0.73) | 0.31 ± 0.46 (0.50) | -0.34 ± 0.50 (0.50) | -1.00 ± 0.86 (0.24) |
| Peak E/A ratio | 0.0 1± 0.01 (0.44) | 0.00 ± 0.00 (0.92) | 0.00 ± 0.00 (0.65) | -0.01 ± 0.01 (0.23) |
| Adjusted for the child’s gestational age at birth, sex, weight and length at birth, age in days at examination and random effect of echocardiographic analyst and month of analysis.  ^1^Calculated by use of Teichholz’ formula. | | | | |

Table S3. Adjusted mean differences comparing left ventricular parameters in infants born to underweight, pre-obese or obese women with those in infants born to women with normal pre-pregnancy BMIs, excluding from the cohort infants born to women with gestational hypertension or preeclampsia.

|  | Mean adjusted difference ± standard deviation (p-value) | | | |
| --- | --- | --- | --- | --- |
|  | Maternal BMI<18.5 (Underweight) | Maternal BMI 25.0-29.9  (Pre-Obese) | Maternal BMI 30.0-34.9  (Obesity class I) | Maternal BMI≥35  (Obesity class II and III) |
| **Left ventricular (LV) structure** | | | | |
| Interventricular septum in end-diastole, IVSd (mm) | 0.01 ± 0.01 (0.69) | -0.01 ± 0.01 (0.21) | 0.00 ± 0.01 (0.76) | -0.01 ± 0.02 (0.71) |
| LV posterior wall, LVPWd (mm) | 0.02 ± 0.01 (0.17) | 0.00 ± 0.01 (0.94) | -0.01 ± 0.01 (0.48) | -0.03 ± 0.02 (0.09) |
| LV internal diameter in end-diastole, LVIDd (mm) | -0.01 ± 0.05 (0.83) | -0.05 ± 0.03 (0.03) | -0.10 ± 0.04 (0.02) | -0.01 ± 0.07 (0.84) |
| LV internal diameter in end-systole, LVIDs (mm) | 0.01 ± 0.04 (0.90) | -0.05 ± 0.02 (0.02) | -0.12 ± 0.04 (0.001) | -0.12 ± 0.05 (0.03) |
| End-diastolic volume, EDV (ml)^1^ | -0.01 ± 0.08 (0.95) | -0.09 ± 0.04 (0.03) | -0.17 ± 0.07 (0.02) | -0.01 ± 0.11 (0.92) |
| End-systolic volume, ESV (ml)^1^ | 0.01 ± 0.04 (0.70) | -0.04 ± 0.02 (0.02) | -0.11 ± 0.03 (0.001) | -0.10 ± 0.05 (0.04) |
| **Systolic function** |  |  |  |  |
| Fractional shortening, FS (%)^1^ | -0.06 ± 0.12 (0.62) | 0.07 ± 0.06 (0.31) | 0.24 ± 0.11 (0.03) | 0.57 ± 0.16 (0.001) |
| Ejection fraction, EF (%)^1^ | -0.09 ± 0.17 (0.59) | 0.12 ± 0.09 (0.19) | 0.34 ± 0.15 (0.02) | 0.77 ± 0.23 (0.001) |
| Stroke volume, SV (ml)^1^ | -0.03 ± 0.06 (0.61) | -0.05 ± 0.03 (0.10) | -0.07 ± 0.05 (0.16) | 0.09 ± 0.08 (0.23) |
| Heart rate, HR (beats per minute) | 0.16 ± 0.68 (0.82) | -0.96 ± 0.35 (0.006) | -0.93 ± 0.60 (0.12) | -3.11 ± 0.91 (0.001) |
| **Diastolic function** |  |  |  |  |
| Mitral valve early peak velocity, MvE (cm/sec) | -0.55 ± 0.43 (0.21) | -0.66 ± 0.22 (0.003) | -0.36 ± 0.38 (0.35) | -1.27 ± 0.57 (0.02) |
| Mitral valve atrial peak velocity, MvA (cm/sec) | -0.29 ± 0.44 (0.50) | -0.28 ± 0.22 (0.20) | 0.04 ± 0.38 (0.91) | -1.01 ± 0.57 (0.08) |
| Mitral valve deceleration time, MV DT (cm/sec) | -0.50 ± 0.99 (0.61) | -0.02 ± 0.50 (0.97) | 0.42 ± 0.86 (0.62) | 2.48 ± 1.29 (0.05) |
| Peak E/A ratio | 0.01± 0.01 (0.48) | 0.01± 0.00 (0.06) | 0.01 ± 0.01 (0.26) | 0.01± 0.01 (0.36) |
| Mothers with gestational hypertension and preeclampsia were identified by use of the following diagnose codes: gestational hypertension: O13.9 preeclampsia: O14.0-O14.2 and O15.0-O15.9. In total 1,260 infants were excluded from the analysis due to their mothers having a diagnosis of either gestational hypertension or preeclampsia.  ^1^Calculated by use of Teichholz’ formula.  Adjusted for the child’s gestational age at birth, sex, weight and length at birth, age in days at examination and random effect of echocardiographic analyst and month of analysis. | | | | |

Table S4. Adjusted mean differences comparing left ventricular parameters in infants born to women aged <25 years, 25-29 years, 35-39 years and ≥40 years with those in infants born to women with aged 30-34 years, excluding from the cohort infants born to women with gestational hypertension or preeclampsia.

|  | Mean adjusted difference ± standard deviation (p-value) | | | |
| --- | --- | --- | --- | --- |
|  | Maternal age <25 years | Maternal age 25-29 years | Maternal age 35-39 years | Maternal age ≥40 years |
| **Left ventricular (LV) structure** | | | | |
| Interventricular septum in end-diastole, IVSd (mm) | 0.01± 0.01 (0.71) | -0.01± 0.01 (0.04) | -0.01± 0.01 (0.20) | -0.01± 0.01 (0.27) |
| LV posterior wall, LVPWd (mm) | 0.01± 0.01 (0.62) | 0.00± 0.01 (0.73) | 0.00± 0.01 (0.56) | 0.00± 0.01 (0.95) |
| LV internal diameter in end-diastole, LVIDd (mm) | 0.00± 0.05 (0.94) | 0.02± 0.02 (0.31) | 0.01± 0.03 (0.58) | -0.16± 0.04 (<0.001) |
| LV internal diameter in end-systole, LVIDs (mm) | 0.01± 0.04 (0.85) | 0.02± 0.02 (0.36) | 0.00± 0.02 (0.98) | -0.10± 0.04 (0.006) |
| End-diastolic volume, EDV (ml)^1^ | -0.03± 0.08 (0.67) | 0.05± 0.04 (0.25) | 0.03± 0.04 (0.43) | -0.25± 0.07 (0.001) |
| End-systolic volume, ESV (ml)^1^ | 0.00± 0.04 (0.91) | 0.02± 0.02 (0.35) | 0.00± 0.02 (0.91) | -0.10± 0.03 (0.002) |
| **Systolic function** |  |  |  |  |
| Fractional shortening, FS (%)^1^ | -0.02± 0.12 (0.86) | 0.00± 0.06 (0.96) | 0.05± 0.07 (0.48) | -0.05 ± 0.11 (0.65) |
| Ejection fraction, EF (%)^1^ | -0.03± 0.17 (0.87) | -0.01± 0.08 (0.95) | 0.06± 0.09 (0.52) | -0.03 ± 0.15 (0.85) |
| Stroke volume, SV (ml)^1^ | -0.03± 0.06 (0.60) | 0.03± 0.03 (0.35) | 0.02± 0.03 (0.41) | -0.15 ± 0.05 (0.003) |
| Heart rate, HR (beats per minute) | 0.36± 0.67 (0.59) | -0.30± 0.33 (0.36) | -0.05± 0.36 (0.88) | -0.03 ± 0.62 (0.96) |
| **Diastolic function** |  |  |  |  |
| Mitral valve early peak velocity, MvE (cm/sec) | -0.71± 0.42 (0.09) | 0.02± 0.21 (0.91) | -0.19± 0.23 (0.40) | -0.53± 0.39 (0.17) |
| Mitral valve atrial peak velocity, MvA (cm/sec) | -0.50± 0.42 (0.23) | 0.06± 0.21 (0.77) | -0.06± 0.23 (0.81) | -0.05± 0.39 (0.90) |
| Mitral valve deceleration time, MV DT (cm/sec) | 0.17± 0.95 (0.86) | 0.31± 0.47 (0.52) | -0.33± 0.52 (0.52) | -0.07± 0.89 (0.13) |
| Peak E/A ratio | 0.01± 0.01 (0.52) | 0.00± 0.00 (0.83) | 0.00± 0.00 (0.52) | -0.01± 0.01 (0.17) |
| Mothers with gestational hypertension and preeclampsia were identified by use of the following diagnose codes: gestational hypertension: O13.9 preeclampsia: O14.0-O14.2 and O15.0-O15.9. In total 1,260 infants were excluded from the analysis due to their mothers having a diagnosis of either gestational hypertension or preeclampsia.  ^1^Calculated by use of Teichholz’ formula.  Adjusted for the child’s gestational age at birth, sex, weight and length at birth, age in days at examination and random effect of echocardiographic analyst and month of analysis. | | | | |

Table S5. Adjusted mean differences comparing left ventricular parameters in infants born to underweight, pre-obese or obese women with those in infants born to women with normal pre-pregnancy BMIs, excluding from the cohort infants in whom the Copenhagen Baby Heart Study identified a congenital heart defect.

|  | Mean adjusted difference ± standard deviation (p-value) | | | |
| --- | --- | --- | --- | --- |
|  | Maternal BMI<18.5 (Underweight) | Maternal BMI 25.0-29.9  (Pre-Obese) | Maternal BMI 30.0-34.9  (Obesity class I) | Maternal BMI≥35  (Obesity class II and III) |
| **Left ventricular (LV) structure** | | | | |
| Interventricular septum in end-diastole, IVSd (mm) | 0.00± 0.1 (0.75) | -0.01± 0.01 (0.40) | -0.01± 0.01 (0.48) | -0.01± 0.02 (0.76) |
| LV posterior wall, LVPWd (mm) | 0.01± 0.02 (0.43) | 0.00± 0.01 (0.95) | -0.01± 0.01 (0.66) | -0.02± 0.02 (0.30) |
| LV internal diameter in end-diastole, LVIDd (mm) | 0.02± 0.05 (0.71) | -0.05± 0.03 (0.04) | -0.11 ± 0.04 (0.01) | -0.03± 0.06 (0.63) |
| LV internal diameter in end-systole, LVIDs (mm) | 0.03± 0.04 (0.43) | -0.04± 0.02 (0.05) | -0.11± 0.04 (0.002) | -0.10± 0.05 (0.05) |
| End-diastolic volume, EDV (ml)^1^ | 0.04± 0.08 (0.61) | -0.08± 0.04 (0.05) | -0.17± 0.07 (0.02) | -0.03± 0.10 (0.78) |
| End-systolic volume, ESV (ml)^1^ | 0.04± 0.04 (0.31) | -0.04± 0.02 (0.07) | -0.10± 0.03 (0.003) | -0.08± 0.05 (0.08) |
| **Systolic function** |  |  |  |  |
| Fractional shortening, FS (%)^1^ | -0.11± 0.13 (0.39) | 0.03± 0.06 (0.62) | 0.17± 0.11 (0.12) | 0.46± 0.16 (0.003) |
| Ejection fraction, EF (%)^1^ | -0.16± 0.17 (0.35) | 0.07± 0.09 (0.43) | 0.25± 0.15 (0.09) | 0.62± 0.21 (0.004) |
| Stroke volume, SV (ml)^1^ | -0.01± 0.06 (0.91) | -0.05± 0.03 (0.12) | -0.08± 0.05 (0.09) | 0.05± 0.07 (0.51) |
| Heart rate, HR (beats per Minute) | -0.05± 0.70 (0.95) | -1.27± 0.35 (<0.001) | -1.13± 0.59 (0.06) | -2.89± 0.86 (0.001) |
| **Diastolic function** |  |  |  |  |
| Mitral valve early peak velocity, MvE (cm/sec) | -0.52± 0.44 (0.24) | -0.70± 0.22 (0.001) | -0.67± 0.37 (0.07) | -1.07 ± 0.53 (0.04) |
| Mitral valve atrial peak velocity, MvA (cm/sec) | -0.38± 0.44 (0.39) | -0.32± 0.22 (0.15) | -0.24± 0.37 (0.51) | -1.29 ± 0.53 (0.01) |
| Mitral valve deceleration time, MV DT (cm/sec) | -0.28± 1.00 (0.78) | 0.23± 0.50 (0.65) | 1.29 ± 0.84 (0.13) | 1.40 ± 1.20 (0.25) |
| Peak E/A ratio | 0.00± 0.01 (0.60) | 0.01± 0.00 (0.04) | 0.01± 0.01 (0.28) | 0.00 ± 0.01 (1.00) |
| Congenital heart defects: Atrial septal defects, ventricular septal defects, bicuspid aortic valve, quadricuspid aortic valve, aortic stenosis, pulmonic stenosis, quadricuspid pulmonic valve, coactation of the aorta, tetraology of Fallot, transposition of the great arteries, atrioventricular septal defects, congenitally corrected transposition of the great arteries, situs inversus, Epsteins anomaly, cardiogenic tumors and supradiaphragmatic totally anomalous pulmonary venous return. In total, 1,696 infants were excluded from the analysis due to presence of congenital heart defects identified by the Copenhagen Baby Heart Study.  ^1^Calculated by use of Teichholz’ formula.  Adjusted for the child’s gestational age at birth, sex, weight and length at birth, age in days at examination and random effect of echocardiographic analyst and month of analysis. | | | | |

Table S6. Adjusted mean differences comparing left ventricular parameters in infants born to women aged <25 years, 25-29 years, 35-39 years and ≥40 years with those in infants born to women with aged 30-34 years, excluding from the cohort infants in whom the Copenhagen Baby Heart Study identified a congenital heart defect.

|  | Mean adjusted difference ± standard deviation (p-value) | | | |
| --- | --- | --- | --- | --- |
|  | Maternal age <25 years | Maternal age 25-29 years | Maternal age 35-39 years | Maternal age ≥40 years |
| **Left ventricular (LV) structure** | | | | |
| Interventricular septum in end-diastole, IVSd (mm) | 0.01± 0.01 (0.51) | -0.01± 0.01 (0.04) | -0.01± 0.01 (0.22) | -0.01± 0.01 (0.38) |
| LV posterior wall, LVPWd (mm) | 0.01± 0.01 (0.54) | 0.00± 0.01 (0.83) | 0.00± 0.01 (0.76) | 0.00± 0.01 (0.94) |
| LV internal diameter in end-diastole, LVIDd (mm) | 0.02± 0.05 (0.65) | 0.03± 0.02 (0.29) | 0.00± 0.03 (0.89) | -0.15± 0.04 (0.001) |
| LV internal diameter in end-systole, LVIDs (mm) | 0.02± 0.04 (0.60) | 0.01± 0.02 (0.52) | 0.00± 0.02 (0.89) | -0.09± 0.04 (0.01) |
| End-diastolic volume, EDV (ml)^1^ | 0.01± 0.08 (0.93) | 0.04± 0.04 (0.30) | 0.02± 0.04 (0.70) | -0.24± 0.07 (0.002) |
| End-systolic volume, ESV (ml)^1^ | 0.01± 0.04 (0.87) | 0.01± 0.02 (0.52) | 0.00± 0.02 (0.96) | -0.09± 0.03 (0.006) |
| **Systolic function** |  |  |  |  |
| Fractional shortening, FS (%)^1^ | -0.01± 0.12 (0.94) | 0.03± 0.06 (0.61) | 0.02± 0.07 (0.73) | -0.06± 0.11 (0.60) |
| Ejection fraction, EF(%)^1^ | -0.01± 0.17 (0.94) | 0.03± 0.08 (0.68) | 0.03± 0.09 (0.74) | -0.04± 0.15 (0.78) |
| Stroke volume, SV (ml)^1^ | 0.00± 0.06 (0.98) | 0.03± 0.03 (0.30) | 0.01± 0.03 (0.71) | -0.15± 0.05 (0.004) |
| Heart rate, HR (beats per minute) | 0.43± 0.67 (0.52) | -0.06± 0.33 (0.87) | 0.14± 0.36 (0.70) | 0.37± 0.62 (0.55) |
| **Diastolic function** |  |  |  |  |
| Mitral valve early peak velocity, MvE (cm/sec) | -0.65± 0.42 (0.12) | 0.09± 0.21 (0.67) | -0.14± 0.23 (0.54) | 0.38± 0.39 (0.34) |
| Mitral valve atrial peak velocity, MvA (cm/sec) | -0.39± 0.42 (0.35) | 0.15± 0.21 (0.47) | -0.08± 0.23 (0.72) | -0.10± 0.39 (0.81) |
| Mitral valve deceleration time, MV DT (cm/sec) | 0.01± 0.95 (0.99) | 0.29± 0.47 (0.54) | -0.31± 0.52 (0.55) | -1.08± 0.89 (0.22) |
| Peak E/A ratio | 0.01± 0.01 (0.49) | 0.00± 0.00 (0.80) | 0.00± 0.00 (0.78) | -0.01± 0.01 (0.32) |
| Congenital Heart Defects: Atrial septal defects, ventricular septal defects, bicuspid aortic valve, quadricuspid aortic valve, aortic stenosis, pulmonic stenosis, quadricuspid pulmonic valve, coactation of the aorta, tetraology of Fallot, transposition of the great arteries, atrioventricular septal defects, congenitally corrected transposition of the great arteries, situs inversus, Epsteins anomaly, cardiogenic tumors and supradiaphragmatic totally anomalous pulmonary venous return. In total, 1,696 infants were excluded from the analysis due to presence of congenital heart defects identified by the Copenhagen Baby Heart Study.  ^1^Calculated by use of Teichholz’ formula.  Adjusted for the child’s gestational age at birth, sex, weight and length at birth, age in days at examination and random effect of echocardiographic analyst and month of analysis. | | | | |

|  | Mean adjusted difference ± standard deviation (p-value) | | | |
| --- | --- | --- | --- | --- |
|  | Maternal BMI<18.5 (Underweight) | Maternal BMI 25.0-29.9  (Pre-Obese) | Maternal BMI 30.0-34.9  (Obesity class I) | Maternal BMI≥35  (Obesity class II and III) |
| **Left ventricular (LV) structure** | | | | |
| Interventricular septum in end-diastole, IVSd (mm) | 0.00± 0.01 (0.90) | -0.01± 0.01 (0.36) | 0.00± 0.01 (0.82) | -0.01± 0.02 (0.60) |
| LV posterior wall, LVPWd (mm) | 0.01± 0.01 (0.48) | 0.00± 0.01 (0.58) | 0.00± 0.01 (0.78) | -0.03± 0.02 (0.09) |
| LV internal diameter in end-diastole, LVIDd (mm) | 0.00± 0.05 (0.93) | -0.05± 0.03 (0.04) | -0.10± 0.04 (0.02) | -0.04± 0.06 (0.54) |
| LV internal diameter in end-systole, LVIDs (mm) | 0.01± 0.04 (0.77) | -0.05± 0.02 (0.03) | -0.12± 0.04 (0.001) | -0.12± 0.05 (0.03) |
| End-diastolic volume, EDV (ml)^1^ | 0.02± 0.08 (0.84) | -0.08± 0.04 (0.05) | -0.16± 0.07 (0.03) | -0.05± 0.11 (0.65) |
| End-systolic volume, ESV (ml)^1^ | 0.02± 0.04 (0.61) | -0.04± 0.02 (0.05) | -0.10± 0.03 (0.002) | -0.10± 0.05 (0.04) |
| **Systolic function** |  |  |  |  |
| Fractional shortening, FS (%)^1^ | -0.05± 0.12 (0.70) | 0.05± 0.06 (0.41) | 0.25± 0.11 (0.02) | 0.47± 0.16 (0.004) |
| Ejection fraction, EF (%)^1^ | -0.07± 0.17 (0.66) | 0.09± 0.09 (0.28) | 0.36± 0.15 (0.01) | 0.63± 0.22 (0.005) |
| Stroke volume, SV (ml)^1^ | -0.01± 0.06 (0.82) | -0.05± 0.03 (0.11) | -0.06± 0.05 (0.21) | 0.04± 0.07 (0.59) |
| Heart rate, HR (beats per minute) | 0.18± 0.67 (0.79) | -1.05± 0.35 (0.003) | -0.89± 0.59 (0.13) | -4.08± 0.90 (<0.001) |
| **Diastolic function** |  |  |  |  |
| Mitral valve early peak velocity, MvE (cm/sec) | -0.45± 0.43 (0.29) | -0.66± 0.22 (0.003) | -0.46± 0.37 (0.21) | -1.25± 0.56 (0.03) |
| Mitral valve atrial peak velocity, MvA (cm/sec) | -0.22± 0.43 (0.61) | -0.34± 0.22 (0.12) | -0.09± 0.37 (0.82) | -0.71± 0.56 (0.20) |
| Mitral valve deceleration time, MV DT (cm/sec) | -0.62± 0.98 (0.53) | 0.09± 0.50 (0.86) | 1.34± 0.84 (0.68) | 1.77± 1.26 (0.16) |
| Peak E/A ratio | 0.01± 0.01 (0.47) | 0.01± 0.00 (0.07) | 0.01± 0.01 (0.23) | 0.01± 0.01 (0.21) |
| Mothers with pregestational and gestational diabetes were identified by use of the following diagnose codes: pregestational diabetes: DO24-DO25, gestational diabetes: DO244. In total 819 infants were excluded from the analysis: 738 infants whose mothers were diagnosed with pregestational or gestational diabetes and 81 infants whose mothers lacked information on diabetes status.  ^1^Calculated by use of Teichholz’ formula.  Adjusted for the child’s gestational age at birth, sex, weight and length at birth, age in days at examination and random effect of echocardiographic analyst and month of analysis. | | | | |

Table S7. Adjusted mean differences comparing left ventricular parameters in infants born to underweight, pre-obese or obese women with those in infants born to women with normal pre-pregnancy BMIs, excluding from the cohort infants born to women with pre-gestational or gestational diabetes.
